# Genotype–phenotype variations in PURA syndrome: Asian and non-Asian perspectives from a systematic review

**DOI:** 10.1186/s13023-025-03908-9

**Published:** 2025-07-25

**Authors:** Shu-Ning Liu, Ching-Shiang Chi, Hsiu-Fen Lee, Chi-Ren Tsai, Yao-Lun Yang, Pei-Yu Wu

**Affiliations:** 1 Division of Pediatric Neurology, Children’s Medical Center, Taichung Veterans General Hospital, 1650, Taiwan Boulevard Sec. 4, Taichung, 407 Taiwan; 2 Department of Post-Baccalaureate Medicine, College of Medicine, National Chung Hsing University, 145, Xingda Rd., Taichung, 402 Taiwan

**Keywords:** Asians, Atrioventricular block, Clinical features, Non-Asians, PURA syndrome

## Abstract

**Background:**

The focus of this study was a comparison of the phenotypical and genotypical differences in PURA syndrome among Asian and non-Asian patients. A retrospective cohort study was performed in a single medical center from January 2014 to May 2025 on patients carrying causative genes for PURA syndrome. A systematic search in PubMed, MEDLINE, Web of Science, and Embase covering the period from January 2014 to May 2025 was conducted. Individuals with PURA syndrome were collected and categorized into Asian and non-Asian groups for analysis. Clinical characteristics, imaging findings, and developmental outcomes were compared between the two groups using Chi-squared or Fisher’s exact tests, with a *p* < 0.05 considered statistically significant.

**Results:**

Of 200 individuals enrolled, 44 were Asian and 156 were non-Asian. 80% or more of individuals with PURA syndrome in both groups shared common clinical features of hypotonia and feeding difficulties during the neonatal period. In terms of neurologic symptoms, there were significantly higher rates of pathological startle response (*p* < 0.01), and lower rates of epilepsy (*p* < 0.01) and movement disorders (*p* = 0.035) among Asian populations. For extra-neurologic symptoms, Asian populations showed a higher incidence of cardiac (*p* = 0.013) and urogenital abnormalities (*p* < 0.01), with statistical significance. A single patient in the cohort study exhibited second-degree atrioventricular block, which required pacemaker placement. Highly heterogeneous variants were identified with 106 causative variants in 200 individuals, including a novel causative variant, c.42_57del (p.Leu15fs), from our cohort. All individuals with PURA syndrome displayed evident psychomotor impairment during follow-up.

**Conclusions:**

PURA syndrome exhibits high phenotypic and genotypic heterogeneity. Increased pathological startle response, reduced epilepsy and movement disorders, and higher rates of cardiac and urogenital abnormalities were observed in the Asian group. Cardiac conduction disorder may prove fatal without timely intervention.

**Supplementary Information:**

The online version contains supplementary material available at 10.1186/s13023-025-03908-9.

## Background

PURA syndrome which comprises PURA-related neurodevelopmental disorders (MIM600473), is caused by causative variants in the *PURA* gene; and 5q31.3 microdeletion syndrome, caused by 5q31.3 microdeletion which may encompass all, or part, of *PURA* [15]. The onset is typically at the neonatal stage with symptoms of hypotonia, hypoventilation, and feeding difficulty [18, 19, 25, 40, 48]. Subsequent neurologic effects lead to developmental delay, epilepsy, and movement disorder starting in childhood; extra-neurological manifestations include cardiovascular, ophthalmic, and skeletal abnormalities in addition to facial dysmorphism exhibited as high anterior hairline, tented upper lip, elongated and unemotional facial expressions [19, 40]. Most individuals present with moderate to severe intellectual disability (ID) and face challenges with independent ambulation or expression of meaningful communication [19]. Besides the inherent symptoms, PURA syndrome phenotypes are heterogeneous with a broad variability of clinical presentations.

PURA syndrome is caused by pathogenic variants in Purine-rich element-binding protein A (*PURA*) gene, which was first identified as the causative variant within the critical deletion segment of 5q31.3 microdeletion syndrome by Lalani et al. in 2014 in 11 individuals with neonatal hypotonia, severe developmental delay with or without epilepsy [25]. PURA syndrome is inherited in an autosomal dominance manner. The *PURA* gene encodes a highly conserved and widely expressed multifunctional protein, Pur-alpha (Purα) [21]. Purα includes N-terminal glycine-rich region, three motifs (PUR I–III) and a C-terminal glutamine–glutamate rich domain. It plays a role in DNA replication, transcription and mRNA trafficking [18, 52] supporting neuronal development of dendrites and axons [17, 23, 40] and expression of the myelin proteolipid protein (Plp1) [15], especially in the cerebellum, cortex, and hippocampus [2, 10, 26]. Additionally, Purα is expressed in various tissues, including muscle, heart, and blood [2, 10, 20, 48], so pathogenic variants in the gene can lead to widespread effects across multiple organ systems.

To date, the majority of large cohort studies on PURA syndrome had been conducted in Western countries with participants primarily recruited from the United States and Europe [18, 19, 26, 40]. Earlier research observed distinct characteristics in Asian individuals compared to those in non-Asian groups [5, 8, 36]. This study aimed to present a case series of PURA syndrome at a single medical center and to conduct a literature review comparing the features of PURA syndrome in Asian and non-Asian populations, with the case series included in the Asian cohort. The comparison revealed statistically significant higher rates of pathological startle response, and lower rates of epilepsy and movement disorders among Asian populations in terms of neurologic symptoms. For extra-neurologic symptoms, Asian populations showed a higher incidence of cardiac and urogenital abnormalities with statistical significance.

## Methods

### Participation and cohort study

#### Participants

For the cohort study and meta-analysis, the inclusion criteria screened for individuals with a disease onset before 18 years of age and a diagnosis of PURA syndrome based on causative gene variants identified via exome sequencing (ES) or genome sequencing (GS). Exclusion criteria screened for individuals with 5q31.3 microdeletion syndrome, where the *PURA* gene was suggested to be causative within the critical deletion region.

#### Cohort study

From January 2014 to May 2025, four individuals at a single medical center with developmental delays, who received ES/GS, and were shown to carry *PURA* gene causative variants, were enrolled. Investigative workups prior to ES/GS comprised family history assessments, developmental milestone evaluations, in-depth physical evaluation and neurological examination such as pathological startle response characterized by jumps of abnormal intensity and/or frequency following unexpected stimuli [14], preliminary laboratory tests, metabolic surveys (including urinary organic acids and tandem mass spectrometry), electroencephalography (EEG), and neuroimaging studies. This study was approved by the Ethics Board of Taichung Veterans General Hospital (TCVGH IRB number: CE20022A and CE22134B).

#### Meta-analysis

A systematic search was conducted on PubMed, MEDLINE, Web of Science, and Embase, adhering to the Preferred Reporting Items for Systematic Reviews and Meta-Analyses (PRISMA) protocols for the duration of the cohort study period, using the terms ''*PURA*'', ''*PURA* gene'', and ''mutation'' and/or ''syndrome''. The study selection process and reasons for exclusion are concisely detailed in supplementary Fig. S1 through the PRISMA flow chart. In the full-text assessment phase, articles were excluded based on the following criteria: full-text unavailable in English, studies lack of formal structure, or insufficient descriptions of clinical data. The aforementioned cohort study of four individuals was included in the analysis.

### Molecular analysis

#### Cohort study

DNA was obtained from each individual's peripheral blood. The NovaSeq 6000 System (Illumina, San Diego, CA, USA) was used to obtain sequencing reads. Our previously published research detailed GS processing, variant calling interpretation, and reporting [27]. The pathogenicity of variants, including pathogenic and likely pathogenic, was determined according to the criteria of the American College of Medical Genetics and Genomics (ACMG) guideline [43].

#### Meta-analysis

Genomic data from all individuals observed in PubMed, MEDLINE, Web of Science, and Embase research was collected for the literature review. Amino acid sequence changes observed in both the cohort study and literature review were annotated with GenBank accession number NM_005859.

### Follow up

#### Cohort study

All individuals received regular neurologic and extra-neurologic examinations. The severity of ID was determined as follows: Individuals younger than 5 years were classified as having mild, moderate, or severe ID if they were 6 months, 7–12 months, or more than 12 months behind normal developmental milestones, respectively. For individuals older than 5 years, cognitive function was assessed using the Wechsler Intelligence Scale for Children-Revised (WISC-R), with scores based on intelligence quotient testing.

#### Meta-analysis

Follow-up results were collected from all individuals identified in the PubMed, MEDLINE, Web of Science, and Embase research.

### Data analysis

#### Cohort study

The medical records of the four recruited individuals were reviewed to collect demographic data, clinical manifestations, neurodevelopmental outcomes, neuroimaging findings and causative gene variants.

#### Meta-analysis

Research data extracted from PubMed, MEDLINE, Web of Science, and Embase included demographic data, clinical characteristics, developmental outcomes, neuroimaging features, and *PURA* gene variants. Individuals were grouped as either Asian or Non-Asian with the cohort study included in the Asian group for analysis. For individuals included in the meta-analysis, ethnicity was extracted from the original publications. In cases where ethnicity was not reported, it was inferred based on the country in which the research was conducted.

Chi-squared tests were used to compare neurologic and extra-neurologic symptoms, psychomotor outcomes and brain magnetic resonance imaging (MRI) features between the two groups. When the expected cell counts were less than five, Fisher’s exact two-sided test was applied to assess statistical significance. A *p* value < 0.05 was considered statistically significant. All statistical analyses were performed using Statistical Product and Service Solutions version 22. Among the articles included in the meta-analysis, some did not provide complete individual-level data. These cases are indicated as ‘‘not applicable” (NA) and were excluded from the statistical analyses conducted for group comparisons.

## Results

### Participants

Recruits totaled 200 individuals with 44 Asians and 156 non-Asians. Tables S1 and S2 summarize demographic data and clinical characteristics of PURA syndrome.

#### Cohort study

Table 1 shows the demographic data, clinical features and investigation results for the cohort study's four individuals diagnosed with PURA syndrome.

**Table 1:** Demographic data, clinical charatcteristics, and causative *PURA* variant of the 4 reported individuals

	Individual 1	Individual 2	Individual 3	Individual 4
Age	13 years old	7 years old	5 years old	8 years old
Gender	Male	Female	Female	Female
Age at Disease onset	Birth	Birth	Birth	Birth
Initial symptoms				
Neurologic symptoms				
Hypotonia	Yes	Yes	Yes	Yes
Extra-neurologic symptoms				
Respiratory distress	Yes	Yes	Yes	Yes
Feeding difficulty	Yes	Yes	No	Yes
Age at first visit	1 year 4 months old	4 months old	1 year old	7 years old
Developmental assessment at first visit				
Gross motor	Head control	Head control	Sit with support	Walk with support
Speech	Phonation	Nil	Phonation	Laugh out
Neurologic symptoms				
Hypotonia	Yes	Yes	Yes	Yes
Startled reaction	Yes	Yes	No	Yes
Seizure	No	No	GTCs at the age of 3 weeks	Myoclonic seizures at 3 months
Extra-neurologic symptoms				
Respiratory distress	Resolved at the age of 2 months	Resolved at the age of 2 years	Resolved at neonatal stage	Resolved at neonatal stage
Feeding difficulty	Resolved at the age of 1 month	Resolved at the age of 6 months	NA	Resolved at the age of 1 year
Cardiac abnormalities	No	PDA, Second degree AV block	No	No
Gastrointestinal abnormalities	No	Drooling	No	Constipation
Ophthalmic abnormalities	Exotropia	Astigmatism, esotropia, horizontal nystagmus	Hyperopia, astigmatism	Hyperopia, astigmatism
Clinical outcome				
Follow up duration	12 years	6 years	4 years	1 year
Psychomotor impairment	Profound	Profound	Profound	Profound
Speech	Nonverbal	Call papa	Nonverbal	Nonverbal
Ambulation	Walk with support	Walk alone	Walk with support	Walk with support
Brain MRI features	Myelination delay, ventriculomegaly	Myelination delay	Myelination delay, ventriculomegaly	Myelination delay, Thin corpus callosum, Mild cerebellar vermis hypoplasia
*PURA* variant (amino acid change)	c.363C > G (p.Tyr121Ter)	c.45_60dup (p.Leu21Glyfs)	c.691_693delTTC (p.Phe233del)	c.42_57del (p.Leu15ProfsTer58)
Inheritance pattern	AD, de novo	AD, de novo	AD, de novo	AD, de novo

AD, autosomal dominant; AV block, atrioventricular block; GTCs, generalized tonic–clonic seizure; NA, not applicable; MRI, magnetic resonance imaging; PDA, patent ductus arteriosus

#### Meta-analysis

In the meta-analysis, the initial search of PubMed, MEDLINE, Web of Science, and Embase yielded 287 records. After removing 45 duplicates, 242 records remained for screening. During the screening phase, 207 articles were excluded for various reasons: 163 were unrelated to the *PURA* gene, 13 discussed the function of the *PURA* gene, 23 mentioned pathogenic variants in the *PURA* gene but lacked individual information and 8 focused on 5q31 microdeletion syndrome instead of PURA syndrome. Finally, our review comprised 12 studies of Asian population (Chinese, Korean, Japanese, Taiwanese, Indian, Vietnamese and Pakistanis) [5, 8, 12, 13, 28, 29, 31, 34, 36, 42, 53, 54], 23 of non-Asian population (American, Australian, British, Brazilian, Columbian, Italian, Portuguese, Tunisian and mixed European) [3, 4, 6, 7, 11, 16, 18, 19, 22, 25, 26, 30, 32, 33, 35, 39–41, 44–46, 48, 49] and 1 of both Asian and non-Asian population (Fig. S1) [38]. 44 Asians and 156 non-Asians were found.

### Phenotype

#### Initial symptoms

**Fig. 1:**
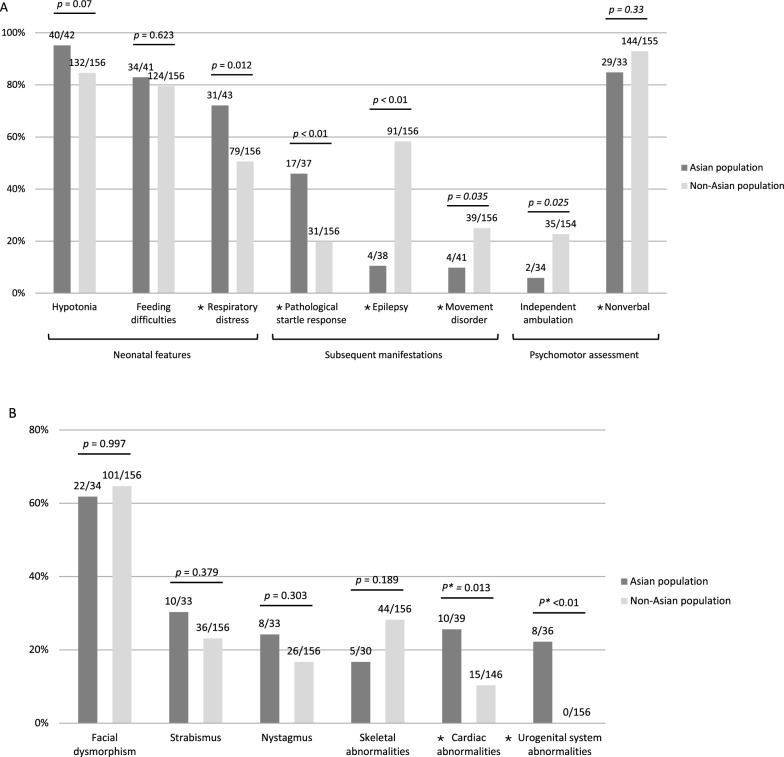
Clinical characteristics of Asian and non-Asian populations. Panel A presents neurologic features at disease onset, subsequent manifestations, and psychomotor assessment at the last follow-up; Panel B showed extra-neurologic symptoms and signs. For each characteristic, the *p* value of comparing the two groups is displayed at the end of each bar. The proportion of affected individuals is expressed as n/N where n is the number of individuals exhibiting the feature and N is the total number of individuals with available data for that characteristic. An asterisk (*) denotes a statistically significant difference (*p* < 0.05)

Figure 1A (Panels 1–3) illustrated symptom presentation in the two groups. At the neonatal stage, hypotonia was present in 95.2% of the Asian individuals and 84.6% of the non-Asian individuals (*p* = 0.07), while feeding difficulties were observed in 82.9% and 79.5% respectively (*p* = 0.623). Respiratory distress was significantly more common in the Asian group at 72.1% compared to the non-Asian group at 50.6% (*p* = 0.012). The Asian group had 14 individuals requiring endotracheal tubes with mechanical ventilation, 4 needing non-invasive ventilation and 4 requiring solely oxygen supplementation. The 79 non-Asians with respiratory distress, required mechanical ventilation or oxygen supplementation for 19 individuals.

#### Neurologic symptoms

Neurologic symptoms for the two groups are shown in Fig. 1A, Panels 4–6. Pathological startle response was detected in 45.9% of Asian individuals and 19.9% of non-Asian populations, respectively. Seizures were documented in 10.5% of the Asian group and 58.3% of the non-Asian group. Seizure patterns in Asian individuals included generalized tonic–clonic seizures (GTCs) and myoclonic seizures with infant stage onset. In two cases, seizures resolved spontaneously without antiseizure medication. In non-Asian individuals, GTC, absence, clonic, myoclonic, tonic seizures and epileptic spasms were described with onset age from birth to 18 years. Movement disorders were observed in 9.8% of the Asian group and 25% in non-Asian group, manifesting as orolingual dyskinesia, choreoathetosis, dystonia, and stereotypical head and hand movements. Bradykinesia and limb rigidity were documented in one individual from Italy [35]. Differences in pathological startle response, seizures, and movement disorders between the Asian and non-Asian groups were all statistically significant (*p* < 0.01, < 0.01, and 0.035, respectively).

#### Extra-neurologic symptoms

Figure 1B illustrates the percentage of extra-neurologic symptoms of the two groups. Facial dysmorphism was observed in 61.8% and 64.7% in Asian and non-Asian populations, respectively. Among Asians, common facial characteristics included long face, tented upper lip, depressed nasal bridge, and telecanthus, while hypotonic facial appearance was notable among non-Asians. A unique case of cutis laxa, characterized by redundant skin folds prominent at thoracic, abdominal, and inferior limbs, was documented in an individual from Italy [6]. Regarding ophthalmic abnormalities, strabismus was reported in 30.3% of Asians and 23.1% of non-Asians, while nystagmus was detected in 24.2% of the Asian group and 16.7% of the non-Asian group. Skeletal abnormalities, including scoliosis, hip dysplasia and hyperlaxity were present in 16.7% of Asian individuals and 28.2% in non-Asian individuals. Hip dysplasia was the most commonly observed skeletal issue among Asians, while scoliosis was most frequently reported in non-Asians.

Cardiac comorbidities were identified in 25.6% of individuals in the Asian group and 10.3% in the non-Asian group, representing a statistically significant difference (*p* = 0.013). Individual 2 in our cohort exhibited significant cardiac abnormalities, beginning on the day 14 of life with a diagnosis of patent ductus arteriosus (PDA) accompanied by symptoms of tachypnea, grunting, and cyanosis. Coil closure was performed on day 34. At age 3, bradycardia with a heart rate ranging from 40 to 90 beats per minute was detected, leading to a diagnosis of second-degree atrioventricular (AV) block, Mobitz Type I, and insertion of a pacemaker. In the literature review, atrial septal defect (ASD), PDA, and AV block were documented in Asian individuals, while ASD, PDA, ventricular septal defect (VSD), left ventricular hypertrophy, and increased QT interval were reported in non-Asian individuals. Urogenital comorbidities were observed exclusively in the Asian group, with an incidence of 22.9%, including cryptorchidism in four individuals and rectovestibular fistula in one individual.

### Genotype

**Fig. 2:**
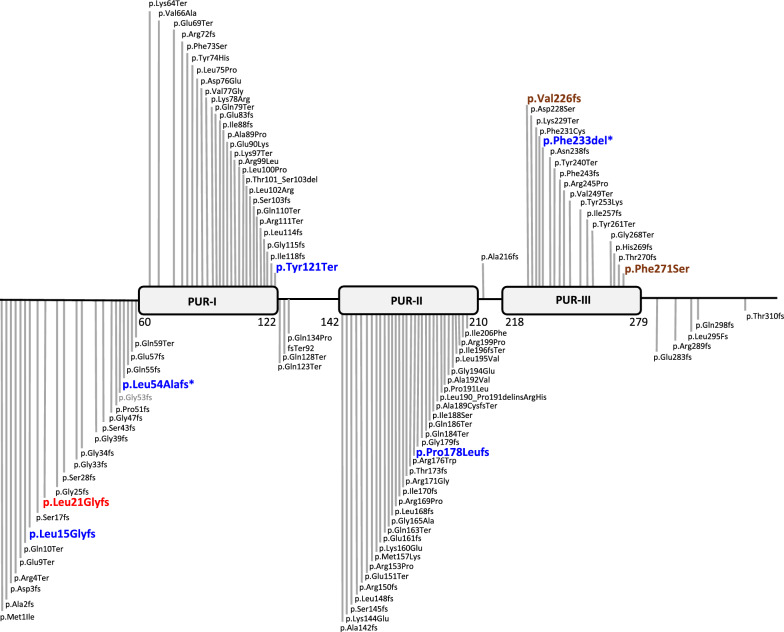
Heterogeneity of *PURA* gene variants. Blue indicates the most five common recurrent *PURA* variants in Asian population, while brown represents the most four common recurrent variants in non-Asian population. A novel *PURA* variant from our case series is highlighted in red, and other variants from this series and the literature review are shown in gray. Protein domains are as follows: PUR-I (60–122), PUR-II (142–210), and PUR-III (222–280). An asterisk (*) denotes the most frequent recurrent variants in both Asian and non-Asian populations

Figure 2 shows *PURA* causative variants. Among 200 individuals, 106 *PURA* variants were identified. All variants occurred de novo, with the exception of a single case in which the variant c.296G > T (p.Arg99Leu) was maternally inherited [16]. The variants were distributed throughout the gene: 25.4% in the PUR-I repeat, 30.2% in the PUR-II repeat, and 16% in the PUR-III repeat, whereas the remainder were outside the PUR repeats. The percentages of frameshift, missense, nonsense, and deletion variants were 45.9%, 25.8%, 23%, and 5.3%, respectively. The most highly recurrent pathogenic variants of *PURA* gene were p.Phe233del, found in 5 Asian and 17 non-Asian individuals; p.Leu54Alafs, observed in 6 Asian and 8 non-Asian individuals; and p.Phe271del, documented in 1 Asian and 4 non-Asian individuals. All other *PURA* causative variants observed in both groups occurred in 4, or fewer, individuals. 12 variants were exclusively seen in the Asian group, while 76 variants were recorded only in the non-Asian group. In the Asian group, p.Leu15Glyfs and p.Pro178Leufs were observed in 2 individuals and all remaining variants were found in only 1 individual. In the non-Asian group, p. Val226fs was observed in 4 individuals, p.Lys97Ter, p.Ile188Ser, p.Arg245Pro, p.Gln128Ter, p.Tyr74His and p.Gln123Ter were observed in 3 individuals and all other variants occurred in 1 or 2 individuals. One individual in our cohort had *PURA* c.42_57del (p.Leu15fs) variant, which has not been previously reported in literature.

### Neuroimaging

**Fig. 3:**
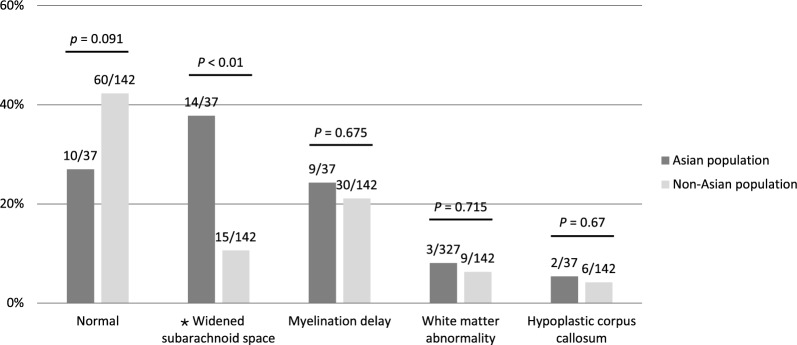
Brain magnetic resonance imaging findings in Asian and non-Asian populations are shown as percentages. For each finding, the *p* value of the comparing the two groups is displayed at the end of each bar. The proportion of affected individuals is expressed as n/N where n is the number of individuals exhibiting the feature and N is the total number of individuals with available data for that characteristic. An asterisk (*) denotes a statistically significant difference (*p* < 0.05)

Figure 3 shows brain MRI findings of the two population groups. Normal brain MRIs were recorded in 27% in the Asian group and 42.3% in the non-Asian group. The two most common anomalies detected were widened subarachnoid space, present in 37.8% of the Asian and 10.6% of the non-Asian population and myelination delay, observed in 24.3% of Asian and 21.1% of non-Asian individuals. A significantly higher frequency of widened subarachnoid space was observed in the Asian population compared to the non-Asian population (*p* < 0.01). Additional findings included white matter abnormality, hypoplastic corpus callosum, and mild hypoplasia of cerebellar vermis, each affecting less than 10% of both groups. In Asian group, one individual displayed a slightly low signal on T1-weighted imaging (T1WI) and slightly hyperintense signal on T2-weighted imaging (T2WI) in the frontal lobe [29]. Follow-up brain MRIs for individuals 3 and 4 in our cohort demonstrated improved myelination.

### Clinical outcome

Figure 1A, panels 7 and 8, show the comparison of the two groups in terms of psychomotor assessment at the last follow-up. All individuals exhibited prominent global developmental delay. Independent ambulation was significantly less common among Asian individuals (5.9%) compared to non-Asian individuals (22.7%) (*p* = 0.025). The median age of sitting alone was 1 year and 7 months in the Asian group and 1 year and 4 months and the non-Asian group. The median age of walking alone was 4 years and 6 months in the Asian group and 3 years and 6 months in the non-Asian group. Notably, 5 non-Asian individuals regressed from walking to being non-ambulatory whereas no regression was observed among Asian individuals. Among those who could ambulate, toe-tip or ataxic gait were frequently observed. At their last assessment, 84.8% of Asian individuals and 92.9% of non-Asian individuals were nonverbal. While the majority of individuals with PURA syndrome exhibited moderate to severe ID, a few exceptions have been reported. One individual from China was noted to have mild ID and was able to attend regular primary school [8]. Kaspi et al. and Hildebrand et al. described a mother and daughter carrying the same *PURA* variant who both demonstrated borderline intellectual functioning and completed mainstream education with classroom support [16, 22].

Early mortality was observed in three individuals in the Asian population. Two of these individuals died of neonatal respiratory failure [8, 54]. The third individual died at 3 months of age due to unknown causes [29]. Early death occurred for four individuals in the non-Asian group. One individual died at 15 years of age old due to sudden unexpected death in epilepsy (SUDEP). Three individuals—one aged 3 and two in their twenties—died following progressive neurologic deterioration resulting in respiratory failure [19].

## Discussion

At disease onset in PURA syndrome, both groups presented common neonatal hypotonia, respiratory distress and feeding difficulties. Experimental studies revealed that homozygous mice lacking both *PURA* alleles (*PURA*−/−) exhibited slack posture, flaccid tail and waddling gait [2, 23] Additionally, heterozygous mice missing one *PURA* allele (+/−) showed limb and trunk hypotonia. Clinically, neonatal onset hypotonia in PURA syndrome may resemble other neurologic disorders [1], such as spinal muscular atrophy [9], congenital myopathies, muscular dystrophies, or Prader-Willi Syndrome. However, PURA syndrome could be distinguished by normal deep tendon reflexes from spinal muscular atrophy or congenital myopathies, normal CK levels without maternal history from myotonic dystrophies, and absence of decreased fetal movement, distinctive facial features, or genital hypoplasia from Prader-Willi Syndrome.

Both groups exhibited distinct neurologic features in addition to shared characteristics. Seizures were significantly more common among non-Asian individuals. Only 10.8% of Asian individuals experienced seizures that resolved spontaneously or with medication, whereas over half of non-Asian individuals developed seizures, with up to two thirds presenting with drug-refractory epilepsy [19]. Treatments included antiseizure medication, vagus nerve stimulation, and Cannabidiol, though no specific regimen was consistently effective [19]. Additional neurological differences included a significantly higher frequency of pathological startle responses among Asian individuals, while movement disorders were significantly more common in the non-Asian group. Although the pathomechanism of seizure has been described in *PURA*(+/−) mice [23]; hyperacusis and movement disorders remain unstudied in *PURA*-related research. Further investigation is needed to clarify the correlation between these clinical features and the role of pur-alpha in the brainstem, corticospinal motor pathways, and basal ganglia [24, 37, 47].

Our study emphasized the importance of cardiac examination in PURA syndrome. Beyond structural heart diseases like PDA, VSD and ASD, the cardiac conduction disorder, second-degree AV block, was identified for the first time. In vivo studies in mice have shown that Purα along Purβ (also belonging to the purine-rich element binding protein family) regulates smooth muscle α-actin gene expression. This combination modulates medial vascular smooth muscle cell function and plays a key role in developmental and pathological heart remodeling [24]. Thus, if respiratory distress persists beyond infancy in PURA syndrome, arrhythmia should be considered. Such comorbidities may emerge later in life, becoming lethal, necessitating pacemaker placement as seen in individual 2 of our cohort, diagnosed with AV block at age 3.

Developmental delay and subsequent ID were evident in nearly all individuals with PURA syndrome, a finding also reflected in animal models. Heterozygous *PURA*( +/−) mice exhibited cognitive and spatial memory deficits, while histopathologic studies of *PURA*(−/−) mice showed reduced cellular proliferation in the cerebellum and hippocampus [23]. Disruption of Purα also led to abnormal oligodendrocytes and hypomyelination [50, 51]. In heterozygous (*PURA* +/−) mice, however, Purα levels steadily increased after birth [23, 31]. In humans, a small proportion of individuals with PURA syndrome achieved ambulation over time, and initial symptoms of respiratory distress and feeding difficulties resolved after infancy in most individuals. Follow-up brain MRI in our cohort similarly indicated improved myelination. Nevertheless, developmental regression was observed in non-Asian individuals with intractable epilepsy [19], suggesting intractable epilepsy may be a poor prognostic factor in PURA syndrome. Our study concluded that epilepsy in PURA syndrome should be actively monitored and treated to prevent developmental regression or SUDEP.

A total of 106 genetic variants were identified in 200 individuals in this study, indicating high heterogeneity in PURA syndrome. Over 70% of these variants were located on PUR repeats, while the novel variant found in our cohort, c.42_57del (p.Leu15fs), was found outside the repeats. In both groups, PUR-II domain contained the most genetic variants, with 34.3% and 28.4% in Asian and non-Asian individuals, respectively. Clinical variability was observed even among variants within the same *PURA* domain, highlighting phenotypic heterogeneity despite pathogenic variants occurring in the same region. The underlying causes of the phenotypic differences between Asian and non-Asian individuals remain unclear. They may involve genetic or environmental modifiers that warrant further investigation. Due to non-specific presentation, multi-organ involvement, and genetic heterogeneity, diagnosing PURA syndrome based on a single clinical characteristic or single gene test is challenging. We recommend early ES or GS for individuals with early-onset hypotonia, respiratory distress and feeding difficulties to identify potential genetic causes. Early detection is crucial, as multi-organ abnormalities, particularly in the cardiac system, may lead to lethal bradycardia, necessitating pacemaker placement.

This analysis faced several limitations. First, clinical information reported in the literature may be incomplete, potentially due to variability in the level of detail provided by authors or differences in diagnostic evaluations among individuals. Additionally, some symptoms may not have manifested at the time of publication, which could lead to underreporting and introduce bias into the statistical analysis. Second, the Asian cohort comprised only one-quarter the size of within the non-Asian cohort, introducing potential sampling bias and likely underestimating heterogenicity in the Asian group. Third, certain clinical characteristics were reported in only one or two individuals, making it difficult to determine whether these findings were attributable to PURA syndrome or incidental.

## Conclusions

Phenotypic and genotypic heterogeneity in PURA syndrome varies widely. A higher prevalence of pathological startle responses, urogenital and cardiac abnormalities was observed in the Asian population, while epilepsy and movement disorders were more common in non-Asians, with all differences reaching statistical significance. Following a diagnosis of PURA syndrome, it is crucial to closely monitor extra-neurologic abnormalities, including ophthalmic, skeletal and cardiac systems, through a multidisciplinary team to manage potential multisystem complications.

## Supplementary Information

Additional file 1

## Data Availability

The data that supports the findings of this study are available in this article.

## Abbreviations

ASD: Atrial septal defect
AV: Atrioventricular
EEG: Electroencephalography
ES: Exome sequencing
GS: Genome sequencing
ID: Intellectual disability
MRI: Magnetic resonance imaging
PDA: Patent ductus arteriosus
PURA: Purine-rich element-binding protein A
Purα: Pur-alpha
SUDEP: Sudden unexpected death in epilepsy
VSD: Ventricular septal defect
