# Supplementary material for: Genotype–phenotype variations in PURA syndrome: Asian and non-Asian perspectives from a systematic review
Source: Orphanet J Rare Dis. 2025 Jul 25;20:376. doi: 10.1186/s13023-025-03908-9 (PMC12297800; doi:10.1186/s13023-025-03908-9)
Supplement: Supplementary file 1 — Additional file 1 [file 13023_2025_3908_MOESM1_ESM.docx]

**
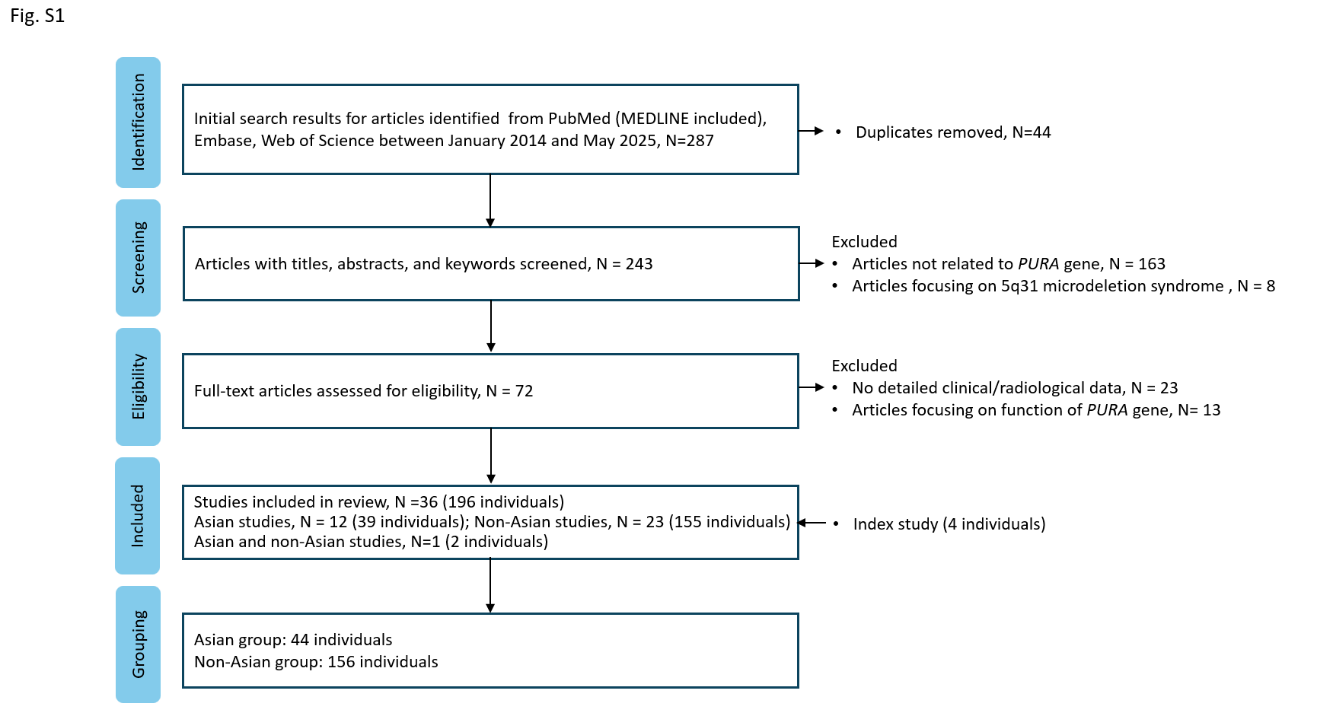
Fig. S1** Study selection process according to PRISMA flow chart

**Table S1**. Demographic data and clinical characteristics of 44 patients in Asian populations.

|  | Dai  et al^15^ | Choi  et al^16^ | Okamoto  et al^17^ | Fukuda  et al^21^ | Noda  et al^22^ | Lin  et al^23^ | Zhang  et al^24^ | Liu  et al^25^ | Wyrebek  et al^26^ | Mishra  et al^27^ | Qashqari  et al^42^ | Richa  et al^28^ | Fujita et al^29^ | Index study | Asian population  n/N (%) |
| --- | --- | --- | --- | --- | --- | --- | --- | --- | --- | --- | --- | --- | --- | --- | --- |
| Patient number | 24 | 5 | 1 | 1 | 1 | 1 | 1 | 1 | 1 | 1 | 1 | 1 | 1 | 4 | 44 |
| Gender, male: female | 11:13 | 4:1 | 1:0 | 0:1 | 1:0 | 1:0 | 1:0 | 1:0 | 0:1 | 0:1 | 1:0 | 0:1 | 0:1 | 1:3 | 22:22 |
| Age at inclusion, median  (range) | 2 yr 3 mo  (1 mo-9 yr) | 4 yr  (3 yr-9 yr) | 10 yr  (nil) | 5 yr  (nil) | 6 yr  (nil) | 10 yr  (nil) | 1 day  (nil) | 4 days  (nil) | 10 mo  (nil) | 4 yr  (nil) | NA  NA | 0yr  (nil) | 7yr  (nil) | 1 yr  (4 mo-7 yr) | 4 yr  (1 day-10 yr) |
| Initial symptoms |  |  |  |  |  |  |  |  |  |  |  |  |  |  |  |
| Neurologic symptom |  |  |  |  |  |  |  |  |  |  |  |  |  |  |  |
| Hypotonia | 21 (/23) | 5 | 1 | 1 | 1 | 1 | 1 | 1 | 1 | 1 | 1 | 1 | 1 | 4 | 40/42 (95.2) |
| Extra-neurologic symptoms |  |  |  |  |  |  |  |  |  |  |  |  |  |  |  |
| Feeding difficulties | 19 (/23) | 2 (/3) | 1 | 1 | 1 | 0 | 1 | 1 | 1 | 1 | 1 | 1 | 1 | 3 | 34/41 (82.9) |
| Respiratory distress | 15 (/23) | 2 | 1 | 1 | 1 | 1 | 1 | 1 | 1 | 0 | 1 | 1 | 1 | 4 | 31/43 (72.1) |
| Subsequent symptoms |  |  |  |  |  |  |  |  |  |  |  |  |  |  |  |
| Neurologic symptoms |  |  |  |  |  |  |  |  |  |  |  |  |  |  |  |
| Pathological startle response | 10 (/20) | 1 | 0 | 1 | 0 | 1 | NA | NA | 0 | 1 | 0 | 0 | NA | 3 | 17/37 (45.9) |
| Epilepsy | 1 (/21) | 0 | 0 | 1 | 0 | 0 | NA | NA | 0 | 0 | 0 | 0 | NA | 2 | 4/38 (10.5) |
| Movement disorder | 0 | 1 | 0 | 0 | 0 | 1 | NA | NA | 0 | 1 | 1 | 0 | NA | 0 | 4/41 (9.8) |
| Extra-neurologic symptoms |  |  |  |  |  |  |  |  |  |  |  |  |  |  |  |
| Facial dysmorphism | 12 (/17) | 2 | 1 | 1 | 1 | 0 | NA | NA | 1 | 1 | 1 | 0 | 1 | 1 | 22/34 (61.8) |
| Ophthalmic abnormalities |  |  |  |  |  |  |  |  |  |  |  |  |  |  |  |
| Strabismus | 3 (/17) | 1 | NA | 1 | 1 | 1 | NA | NA | 0 | 0 | 0 | 0 | NA | 3 | 10/33 (30.3) |
| Nystagmus | 3 (/17) | 2 | NA | 0 | 0 | 0 | NA | NA | 1 | 1 | 0 | 0 | NA | 1 | 8/33 (24.2) |
| Cardiac abnormalities | 8 (/22) | 0 | 0 | 0 | 0 | 0 | NA | NA | 0 | 0 | 1 | 0 | NA | 1 | 10/39 (25.6) |
| Urogenital abnormalities | 6 (/19) | 0 | 0 | 1 | 0 | 0 | NA | NA | 0 | 0 | 0 | 0 | NA | 1 | 8/36 (22.2) |
| Skeletal abnormalities | 3 (/13) | 0 | 0 | 1 | 1 | 0 | NA | NA | 0 | 0 | 0 | 0 | NA | 0 | 5/30 (16.7) |
| Psychomotor outcome§ |  |  |  |  |  |  |  |  |  |  |  |  |  |  |  |
| Independent ambulation | 1 (/19) | 0 | 0 | 0 | 0 | 0 | NA | NA | NA | 0 | NA | NA | 0 | 1 | 2/34 (5.9) |
| Nonverbal | 16 (/18) | 5 | 1 | 1 | 1 | 1 | NA | NA | NA | 0 | NA | NA | 1 | 3 | 29/33 (84.8) |
| Brain MRI features |  |  |  |  |  |  |  |  |  |  |  |  |  |  |  |
| Normal | 3 (/18) | 0 | 1 | 1 | 1 | 1 | 0 | 0 | 1 | 1 | 0 | 1 | NA | 0 | 10/37 (27) |
| Abnormal | 15 (/18) | 5 | 0 | 0 | 0 | 0 | 1 | 1 | 0 | 0 | 1 | 0 | NA | 4 | 27/37 (73) |
| Widened subarachnoid space | 8 (/18) | 5 | 0 | 0 | 0 | 0 | 1 | 0 | 0 | 0 | 0 | 0 | NA | 0 | 14/37 (37.8) |
| Myelination delay | 3 (/18) | 2 | 0 | 0 | 0 | 0 | 0 | 0 | 0 | 0 | 0 | 0 | NA | 4 | 9/37 (24.3) |
| White matter abnormality | 1 (/18) | 0 | 0 | 0 | 0 | 0 | 0 | 1 | 0 | 0 | 1 | 0 | NA | 0 | 3/37 (8.1) |
| Hypoplastic corpus callosum | 0 (/18) | 0 | 0 | 0 | 0 | 0 | 0 | 1 | 0 | 0 | 0 | 0 | NA | 1 | 2/37 (5.4) |

§, age at last follow-up; mo: month; MRI, magnetic resonance imaging; NA, not applicable; yr: year.

**Table S2**. Demographic data and clinical characteristics of 156 patients in non-Asian populations

|  | Johannesse et al^6^ | Mroczek et al^30^ | Cinquina et al^31^ | Nogueira et al^32^ | Qashqari et al^42^ | Spangenberg et al^33^ | Solazzi et al^34^ | Ben et al^35^ | Mora-Martinez et al^36^ | Falsaperla et al^43^ | Crippa, A. C. et al^44^ | Michael S et al^45^ | Sandra et al^47^ | Non-Asian populations n/N (%) |
| --- | --- | --- | --- | --- | --- | --- | --- | --- | --- | --- | --- | --- | --- | --- |
| Patient number | 141 | 3 | 1 | 1 | 1 | 1 | 1 | 1 | 1 | 1 | 1 | 2 | 1 | 156 |
| Gender, male: female | 57:84 | 2:1 | 1:0 | 0:1 | 0:1 | 1:0 | 1:0 | 0:1 | 0:1 | 1:0 | 1:0 | 0:2 | 1:0 | 65:91 |
| Age at inclusion, median  (range) | 6 yr  (5 mo-48 yr) | 9 yr  (2-12 yr) | 3 yr  (nil) | 9 yr  (nil) | 5 yr  (nil) | 6 yr  (nil) | 7 yr  (nil) | 2.8 yr  (nil) | 7 yr  (nil) | 11 yr  (nil) | 7 yr  (nil) | 25.5 yr  (13-38yr) | 7 yr  (nil) | 7 yr  (5 mo-48 yr) |
| Initial symptoms |  |  |  |  |  |  |  |  |  |  |  |  |  |  |
| Neurologic symptoms |  |  |  |  |  |  |  |  |  |  |  |  |  |  |
| Hypotonia | 120 | 2 | 1 | 1 | 1 | 1 | 1 | 1 | 0 | 1 | 1 | 1 | 1 | 132/156 (84.6) |
| Extra-neurologic symptoms |  |  |  |  |  |  |  |  |  |  |  |  |  |  |
| Feeding difficulties | 114 | 1 | 1 | 1 | 1 | 1 | 1 | 1 | 0 | 1 | 1 | 0 | 1 | 124/156 (79.5) |
| Respiratory distress | 75 | 0 | 1 | 0 | 1 | 0 | 0 | 0 | 0 | 0 | 0 | 1 | 1 | 79/156 (50.6) |
| Subsequent symptoms |  |  |  |  |  |  |  |  |  |  |  |  |  |  |
| Neurologic symptoms |  |  |  |  |  |  |  |  |  |  |  |  |  |  |
| Pathological startle response | 25 | 0 | 1 | 1 | 1 | 0 | 1 | 1 | 0 | 0 | 1 | 0 | 0 | 31/156 (19.9) |
| Epilepsy | 84 | 0 | 0 | 1 | 0 | 1 | 1 | 1 | 0 | 1 | 1 | 0 | 1 | 91/156 (58.3) |
| Movement disorder | 34 | 0 | 0 | 1 | 0 | 1 | 1 | 1 | 0 | 0 | 1 | 0 | 0 | 39/156 (25) |
| Extra-neurologic symptoms |  |  |  |  |  |  |  |  |  |  |  |  |  |  |
| Facial dysmorphism | 91 | 1 | 1 | 1 | 0 | 1 | 1 | 1 | 0 | 0 | 1 | 2 | 1 | 101/156 (64.7) |
| Ophthalmic abnormalities |  |  |  |  |  |  |  |  |  |  |  |  |  |  |
| Strabismus | 33 | 0 | 0 | 1 | 0 | 0 | 0 | 0 | 1 | 0 | 0 | 0 | 1 | 36/156 (23.1) |
| Nystagmus | 24 | 0 | 0 | 0 | 0 | 0 | 1 | 1 | 0 | 0 | 0 | 0 | 0 | 26/156 (16.7) |
| Cardiac abnormalities | 15 (/133) | 0 | 0 | 0 | 0 | 0 | 0 | 0 | 0 | 0 | 0 | 0 | 0 | 15/146 (10.3) |
| Urogenital abnormalities | 0 | 0 | 0 | 0 | 0 | 0 | 0 | 0 | 0 | 0 | 0 | 0 | 0 | 0/156 (0) |
| Skeletal abnormalities | 39 | 1 | 0 | 1 | 0 | 0 | 0 | 0 | 1 | 0 | 1 | 0 | 1 | 44/156 (28.2) |
| Psychomotor outcome§ |  |  |  |  |  |  |  |  |  |  |  |  |  |  |
| Independent ambulation | 28 | 2 | 0 | 1 | NA | 0 | 1 | NA | 0 | 0 | 0 | 2 | 1 | 35/154 (22.7) |
| Nonverbal | 132 | 3 | 1 | 1 | NA | 1 | 1 | 1 | 1 | 1 | 1 | 0 | 1 | 144/155 (92.9) |
| Brain MRI features |  |  |  |  |  |  |  |  |  |  |  |  |  |  |
| Normal | 56 (/129) | 0 | 0 | 0 | NA | 1 | 0 | 0 | 0 | 0 | 1 | 2 | NA | 60/142 (42.3) |
| Abnormal | 73 (/129) | 1 | 1 | 1 | NA | 0 | 1 | 1 | 1 | 1 | 0 | 0 | NA | 80/142 (56.3) |
| Widened subarachnoid space | 13 (/129) | 0 | 0 | 1 | NA | 0 | 0 | 0 | 1 | 0 | 0 | 0 | NA | 15/142 (10.6) |
| Myelination delay | 25 (/129) | 1 | 1 | 1 | NA | 0 | 1 | 1 | 0 | 0 | 0 | 0 | NA | 30/142 (21.1) |
| White matter abnormality | 8 (/129) | 1 | 0 | 0 | NA | 0 | 0 | 0 | 0 | 0 | 0 | 0 | NA | 9/142 (6.3) |
| Hypoplastic corpus callosum | 5 (/129) | 0 | 0 | 0 | NA | 0 | 0 | 0 | 0 | 1 | 0 | 0 | NA | 6/142 (4.2) |

§, age at last follow-up; mo: month; MRI, magnetic resonance imaging; NA, not applicable; yr: year.
